# Bluetooth Low Energy Mesh: Applications, Considerations and Current State-of-the-Art

**DOI:** 10.3390/s23041826

**Published:** 2023-02-06

**Authors:** Iynkaran Natgunanathan, Niroshinie Fernando, Seng W. Loke, Charitha Weerasuriya

**Affiliations:** 1 School of Information Technology, Deakin University, Geelong, VIC 3220, Australia; 2 Nanotechnology Facility, Swinburne University of Technology, John St., Hawthorn, VIC 3122, Australia; 3 Ampcontrol Burn Brite Ltd., 1/100 New Street, Ringwood, VIC 3134, Australia

**Keywords:** Bluetooth Mesh, Bluetooth, networking, communication

## Abstract

With the proliferation of IoT applications, more and more smart, connected devices will be required to communicate with one another, operating in situations that involve diverse levels of range and cost requirements, user interactions, mobility, and energy constraints. Wireless technologies that can satisfy the aforementioned requirements will be vital to realise emerging market opportunities in the IoT sector. Bluetooth Mesh is a new wireless protocol that extends the core Bluetooth low energy (BLE) stack and promises to support reliable and scalable IoT systems where thousands of devices such as sensors, smartphones, wearables, robots, and everyday appliances operate together. In this article, we present a comprehensive discussion on current research directions and existing use cases for Bluetooth Mesh, with recommendations for best practices so that researchers and practitioners can better understand how they can use Bluetooth Mesh in IoT scenarios.

## 1. Introduction

Communication plays a vital role in many modern applications, especially in the area of the Internet-of-Things (IoT). Compared to the traditional wired communication mechanisms, wireless communication technologies are required due to the advantages they offer, including efficiency, availability, flexibility, reduced installation problems, and cost savings. However, wireless technologies, namely ZigBee, Z-Wave, Thread, 6LoWPAN, and Wi-Fi, suffer from drawbacks such as reduced coverage, latency, and low level of reliability due to the loss of data packets, and hence are not able to sufficiently fulfill the requirements of many IoT applications.

Bluetooth Mesh (BT Mesh) is a new wireless protocol officially released in 2017, developed to provide extended coverage and higher robustness as well as reduced cost and low power consumption. This version of Bluetooth is built on top of the core Bluetooth low energy (BLE) stack of the Bluetooth 4.2 specification. BT Mesh allows for many-to-many communication over BLE, enabling the the creation of a mesh network of Bluetooth devices. The features of BT Mesh make it ideally suited for many IoT based applications such as home and industrial automation. Other mesh networks, such as WiFi, ZigBee, Thread, and Z-Wave, use high delay routing techniques, whereas BT Mesh uses a flooding technique that is highly reliable and easy to implement. One of the main disadvantages of flooding is that it usually tries to create a large number of duplicate data packets. To minimize this problem, BT Mesh uses “managed” flooding.

As BT Mesh is an emerging and evolving technology, we have a limited number of papers in the literature that cover various aspects of BT Mesh. There are only a few survey papers focusing on BT Mesh explicitly. For example, the paper in ref. [1] is one of the first papers to discuss the BT Mesh standard and uses a three-pronged approach to evaluate the BT Mesh specification—namely, an experimental evaluation, a statistical approach, and a graph-based simulation using metrics such as round-trip time (RTT), throughput, and energy consumption. However, since this paper was published in 2018, not many related papers using the BT Mesh specification are included. The survey by Yin et al. [2] is the first to review BT Mesh, and it discusses the evolution of BT technology and introduces the new features of Bluetooth 5.0 and BT Mesh. Because many of the new enhancements in BT 5.0 and BT Mesh are for BLE, and hence are similar, the article takes the viewpoint that both specifications are in the same group but fall to different layers of upgrades. This article was published in 2019, and BT Mesh had only been officially released by the Bluetooth Special Interest Group (SIG) in 2017. Hence, very few related papers on BT Mesh are reviewed in [2]. The survey paper by Ghori, Wan, and Sodhy in 2020 [3] reviews BLE-based mesh network protocols and related security issues. However, this article’s focus is not on the BT Mesh standard itself and considers various BLE-based mesh communication protocols. The survey paper by Hernández-Solana et. al [4] provides an overview of the BT Mesh standard with an analysis of the BT Mesh technology and discusses associated challenges. The authors discuss technology related to BT Mesh in detail, focusing on BT Mesh architecture and the illustration of BT Mesh communication protocol. In [5], Darroudi, Gomez, and Crowcroft provide a comprehensive comparison of two BLE mesh networking standards, BT Mesh and 6BLEMesh, produced by the Bluetooth SIG and IETF, respectively, discussing their main features and performance characteristics and trade-offs.

However, none of these existing survey papers comprehensively consider the heterogeneous requirements of various IoT scenarios and discuss how BT Mesh can be applied in practice in diverse conditions. To the best of our knowledge, there is no single BT Mesh related paper that can serve as a reference point for a practitioner to understand its architecture, how it compares with other related wireless protocols, what has been investigated in related BT Mesh literature, and how it can be used in IoT scenarios. This paper aims to address this gap. The primary focus of this paper is to provide a comprehensive overview of BT Mesh that includes a brief introduction of BT Mesh technology, a comparison with other wireless technologies such as Wi-Fi, Z-Wave, and Zigbee, and a discussion about the current implementations of BT Mesh that are reported in the literature with an analysis of IoT application requirements and how they can be fulfilled by BT Mesh. We have outlined a number of different aspects of BT Mesh, such as reliability, delay, scalability, memory utilization, and effects on user traffic. Further, we have implemented a basic BT Mesh network and presented our observations. As BT Mesh technology is in its early days, we also discuss the challenges related to BT Mesh technology and its future directions.

The main contributions of the paper can be summarised below.

- To the best of our knowledge, this is the first research work exploring the practical applicability, challenges, and opportunities of using BT Mesh for a diverse set of IoT scenarios.
- This work presents a review of BT Mesh experimental investigations from related work and insights from our test-bed, analysing real-world implications for latency, energy, scalability, and reliability based on BT Mesh parameters and behaviour.

In this study, we used a literature survey design to identify related work on BT Mesh and the Internet of Things. This study reviews research that has been published between 2017 and 2022, since the official BT Mesh was released by the Bluetooth Special Interest Group (SIG) in 2017. However, a few papers prior to 2017 have also been included for discussing IoT and software aspects. Digital tools and databases such as Google Scholar, ACM DL, and IEEE Xplore were used to find relevant articles. Articles that focus on Bluetooth without investigation of the BT Mesh standard were excluded.

The remainder of this paper is organized as follows. The overview of the BT Mesh technology with protocol stack, different node types, and privacy and security are presented in Section 2. In Section 3, real-world implementations of BT Mesh in IoT contexts are discussed. The comparison of BT Mesh technology with other related protocols is presented in Section 4. In Section 5, BT Mesh experiments reported in the literature are discussed. The group messaging related experiments performed by us are reported together with the experimental setup in Section 6. The challenges related to BT Mesh are discussed in Section 7. Finally, Section 8 concludes the paper with some future directions.

## 2. Overview of Bluetooth Mesh

The Bluetooth mesh specification was published by the Bluetooth Special Interest Group (SIG) (https://www.bluetooth.com/ accessed on 15 January 2022) in 2017. BT Mesh protocol stack operates on the core Bluetooth low energy (BLE) stack of the Bluetooth^®^ 4.2 specification, which facilitates backward compatibility with Bluetooth version 4.0 and higher.

**Figure 1:**
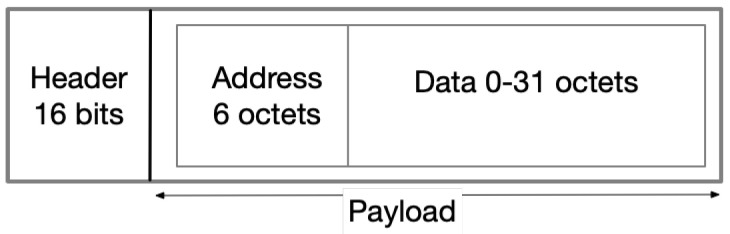
The PDU format of BT Mesh protocol.

The Bluetooth Mesh messages are typically carried inside the payload of BLE advertisement packets (except in the case of legacy Bluetooth devices, which use a different approach, explained later in this section). From the different types of advertising events available in BLE, BT Mesh uses an event type named “nonconnectable undirected event”, which is intended to allow a scanner to receive information from the advertiser, but not to respond to other requests [6]. The corresponding PDU packet format is shown in Figure 1.

**Figure 2:**
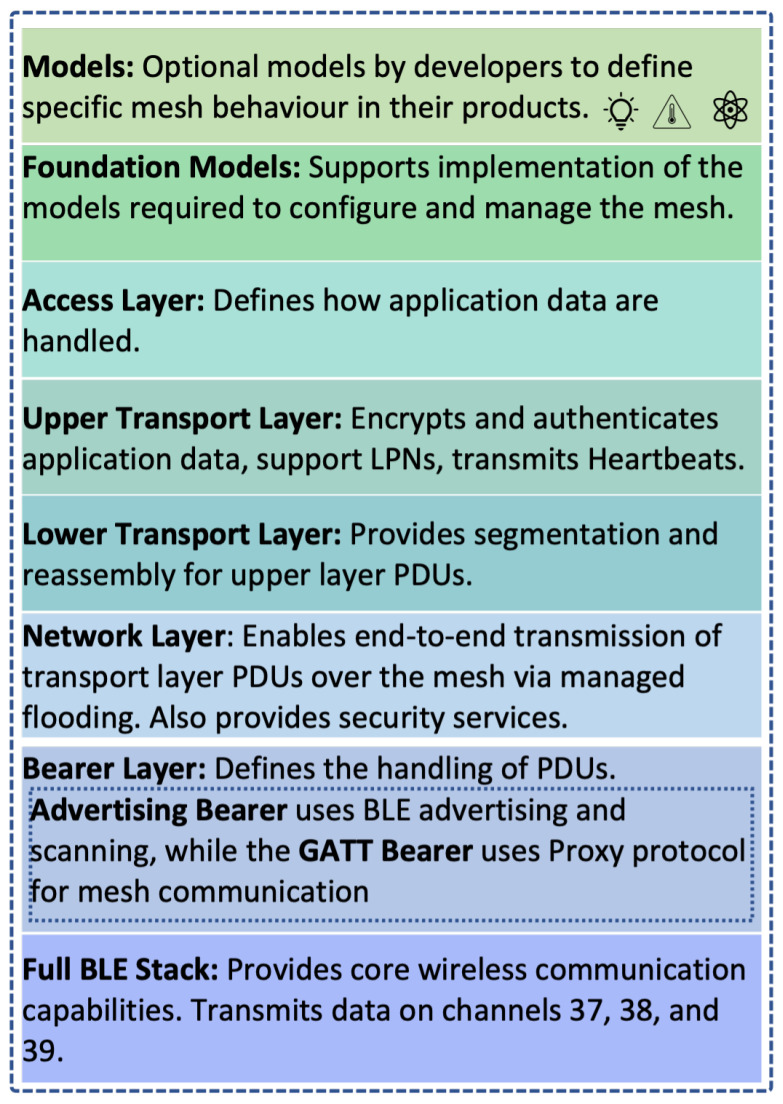
BT Mesh protocol stack.

Figure 2 illustrates the layered architecture of BT Mesh and functions of each layer.

The BLE stack is the foundation of BT Mesh protocol stack, ensuring core wireless communication capabilities. The entire BT Mesh technology works on three advertising channels only, transmitting data on channels 37, 38, and 39 reserved for all connection-less communications.

Data transmission is enabled via the Bearer Layer, which defines how packet data units (PDU) should be handled when they are transmitted. Currently BT Mesh has two bearers defined, namely, Advertising Bearer and Generic Attribute Profile (GATT) Bearer. The Advertising Bearer is the default bearer and defines non-connectable advertising PDUs and scanning configuration to assist mesh communications. When a device is unable to use the Advertising Bearer (such as a legacy Bluetooth low energy device that does not support the Advertising Bearer), the GATT Bearer is used. It utilizes proxy protocol to transmit and receive proxy PDUs in the mesh network. In this way, legacy Bluetooth devices, such as devices with versions older than Bluetooth 4.0, can be allowed to participate in the mesh network. The proxy feature is described in more detail in Section 2.1.4.

The Network Layer helps transport layer PDUs to be transported by the Bearer Layer by defining message address types and a network message format. End-to-end transmission is enabled via a controlled flooding (also called managed flooding) mechanism. Each message header contains a time to live (TTL) value, which dictates the maximum number of hops. The TTL value is decremented each hop. Thus, the TTL field provides a way to “control” the degree of flooding that can take place in the BT Mesh network.

The Lower Transport Layer passes the PDUs it gets from the Upper Transport Layer to the Lower Transport Layer on a peer device. If necessary, this layer has the capability to divide and reorganize the PDUs.

The main purpose of the Upper Transport Layer is to provide security and validation of application data that are exchanged with the Access Layer. It also enables the periodic transmission of heartbeat messages, which signal that the transmitting nodes are still active. They can also be used by receiving nodes to determine how far the sender is [7].

The Access Layer facilitates applications to use the aforementioned Upper Transport Layer and defines the format of application data and encryption and decryption processes. The Access Layer provides standardized communication between different hardware and different device vendors. This was achieved in the Bluetooth Mesh specification by introducing the Element and Model concept.

The top two layers are the Foundation Models and the Models layers. In BT Mesh, models are the collection of concepts, definitions, and functionalities related to the mesh network. Models determine the functions provided by a node. Each node can have multiple models. A model is always part of an Element, which is an addressable entity within a node. In a device there can be one or more elements. Each Element in the device has been assigned a unique unicast address during the provisioning process. For example, a node can contain a model that provides a light switch function as well as another model that provides a sensor data collection function [8]. These models are classified into server model, client model, and control model. The Foundation Model Layer enables the implementation and maintenance of the mesh network. The Model Layer enables implementation of models required for specific BT Mesh applications, such lighting apps for home automation.

### 2.1. Different Node Types in BT Mesh

A BT Mesh network typically includes a large number of devices called nodes. Devices that are not part of the BT Mesh network are called “unprovisioned devices”. The mechanism that converts an unprovisioned device into a node is called “provisioning”. All nodes have the ability to transmit and receive mesh messages. However, based on the additional features, they can be broadly grouped into four types: Relay, Proxy, Friend, and Low Power nodes (LPN) [9].

#### 2.1.1. Relay Node

Nodes that can retransmit received messages are called relay nodes. Relay nodes provide enhanced BT Mesh network coverage area. The mesh coverage area can be extended via relay nodes, as messages can make multiple “hops” between devices by being relayed. However, relay nodes can cause network congestion. To overcome this, in BT Mesh, PDUs include a time to live (TTL) field. The number assigned to this field limits the maximum number of hops away from the originating node a massage can be relayed.

#### 2.1.2. Low-Power Node (LPN)

As the name implies these, LPNs consume lower energy for their functionality. This is achieved via reduction in reception duty cycles. Usually these nodes are battery powered. A typical LPN mostly transmits periodically and only receives data occasionally. Hence LPNs need collaboration of another node to support its functionality.

#### 2.1.3. Friend Node

A node that supports an LPN is known as a friend node. The Friend node stores messages addressed to the LPN and delivers them to the LPN when the LPN requests the messages. When the LPN requests, all the stored messages are forwarded to the LPN sequentially. As friend nodes require a higher amount of energy; they are usually powered by the main AC power supply.

#### 2.1.4. Proxy Node

Currently, certain devices, such as some mobile phones and Wi-Fi-Bluetooth Mesh gateways, can support Bluetooth low energy, but may not be able to support the new BT Mesh networking stack. However, because they support Bluetooth low energy, it is possible for them to participate in a BT Mesh network using the GATT bearer with the help of a Proxy node. A Proxy node functions as a middle entity to assist such legacy devices to connect to the mesh network by exposing the GATT proxy protocol PDUs, and the Proxy node converts them to mesh PDUs. In this way, the Proxy feature helps a node to pass through messages between the GATT and Advertising Bearers. In a typical BT Mesh network, multiple nodes can support the proxy feature.

### 2.2. Addressing

Bluetooth Mesh addressing plays a vital role in the mesh devices and groups identification and communication process. There are three types of addresses in BT Mesh:

1. Unicast addresses: The unicast address is assigned to each element of the node during the new node adding process to the mesh network. The unicast address cannot be changed during the lifetime of the node on the mesh network. The unicast addresses are assigned sequentially.
2. Group addresses: Group addresses are multicast addresses on the mesh network. They are used to communicate with multiple nodes at once. The nodes or elements are assigned to the group address during the node configuration process.
3. Virtual addresses: Users can generate virtual addresses, and they can be considered as a special kind of group address.

### 2.3. Privacy and Security of BT Mesh

To protect the privacy of the data being exchanged, all the messages in BT Mesh are encrypted and authenticated. In BT Mesh, security is handled in different layers: network security, application security, and device security.

Whereas in BLE, GATT devices’ security features are optional, in BT Mesh, security is mandatory. In the BLE GATT devices, the network designer can decide the level of security required for particular applications based on their risk assessment. In BT Mesh, security is enforced, as the security of the entire network is considered instead of individual devices.

#### 2.3.1. Separation of Concerns

The idea behind this feature is to ensure only nodes that need particular information will know the information. In other words, a node could relay a message without knowing the full content of the message. In BT Mesh networking, separate security keys called AppKeys for different applications, such as lighting and heating, are used to secure messages at the network layer. Different network keys are assigned to different subnets. Knowing a network key makes a node member of the corresponding subnet. Using the network keys, network encryption keys and privacy keys are generated. In addition, each node in a BT Mesh network has a unique device key that is used for the configuration and provisioning of the node.

#### 2.3.2. Area Isolation

Each subnet in the mesh can be identified using its unique network key. Hence, access to the subnets can be properly manged within subnets, which cryptographycally isolate individual subnets.

#### 2.3.3. Secure Device Provisioning

Adding a new device without considering security can create serious security problems, as a new device will get the network key that all the other devices in the network have. Therefore, new devices are added to the BT Mesh network via a secure process. Using the instructions provided by the new device manufacturer, the provisioner adds the device to the BT Mesh network. The device addition process involves several steps, including exchange of public keys, authentication, and distribution of the provisioning data. To protect the BT Mesh network from eavesdroppers, an asymmetric cryptography based algorithm is used to create public keys. The authentication is performed via the exchange of cryptographic hash between the device and the provisioner. For the secure distribution of the provisioning data, session keys are used. These session keys are derived from using the respective private keys of two devices.

#### 2.3.4. The Key Refresh Procedure

To enhance the security of the BT Mesh networks, all the security keys are changed on the request of the user. Using the configuration messages, the provisioner generates and sends new security keys to all the nodes in a BT Mesh network. It should be noted that LPNs in a BT Mesh network receive the new keys with a delay. This is due to the fact that they need to receive the keys from their respective “friend” nodes. As all the nodes do not receive their new set of keys at the same time, to ensure proper functioning of the network, a transition period is introduced where both the old keys and the new keys are used. At the end of the transition period, the provisioner informs the nodes that the new key updating is completed.

#### 2.3.5. Message Obfuscation

To protect the privacy of the data, it is important to make sure that the messages are not tracked. To make the tracking of network PDUs difficult, network keys are used to obfuscate network PDU header values.

## 3. Applications of Bluetooth Mesh

BT Mesh network implementations for IoT applications found in the literature can be categorised as according to their use case, as shown in Table 1. Note that “smart buildings” and “smart lighting” have been classified as two categories, as lighting solutions using BT Mesh are not limited to be inside buildings only and can also be outdoors, such as street lighting, underground mines, etc. Furthermore, although there exists a lot of examples using BLE in IoT applications, especially in smart buildings [10,11], a vast majority of these use BLE only and not BT Mesh, and hence have not been considered in this review.

### 3.1. Selected IoT Application Implementations

In this section, we discuss details of practical implementations of selected IoT applications from each category identified in Table 1. We use the term “central node” to indicate a node that receives data from others. The term “server node” is used for a node that is connected to the Internet. It can be assumed that both the central node and server node have more processing capabilities than other nodes. Other terms, such as relay and proxy nodes, are used in their usual meanings, which are defined in Section 2.

#### 3.1.1. Smart Buildings

The concept of smart buildings encompasses features such as energy efficiency, building automation, and various assisted living functions [32]. Localisation and tracking of occupants is required for many intelligent functions provided by smart buildings [33]. Several papers have investigated the feasibility of employing BT Mesh for enabling localisation [12,13,14], with mixed results. For example, in [12], the authors tested an experimental setup consisting of ten anchor nodes (ANs) that were already aware of their location and two relays over two levels in a building. Bluetooth beacons were used as unlocated devices (UDs), periodically advertising messages. The ANs receive, process, and forward messages from the UDs and forward them to a gateway via the mesh network. The gateway then forwards the messages to a remote server that runs the inferencing algorithms necessary for localisation. The authors note that, because the mesh network seemed susceptible to various interference in the environment, careful configuration was needed in the positioning of the nodes. Moreover, the limited number of PDUs per second was also not ideal for transmitting the number of measurements needed for continuous indoor positioning.

**Figure 3:**
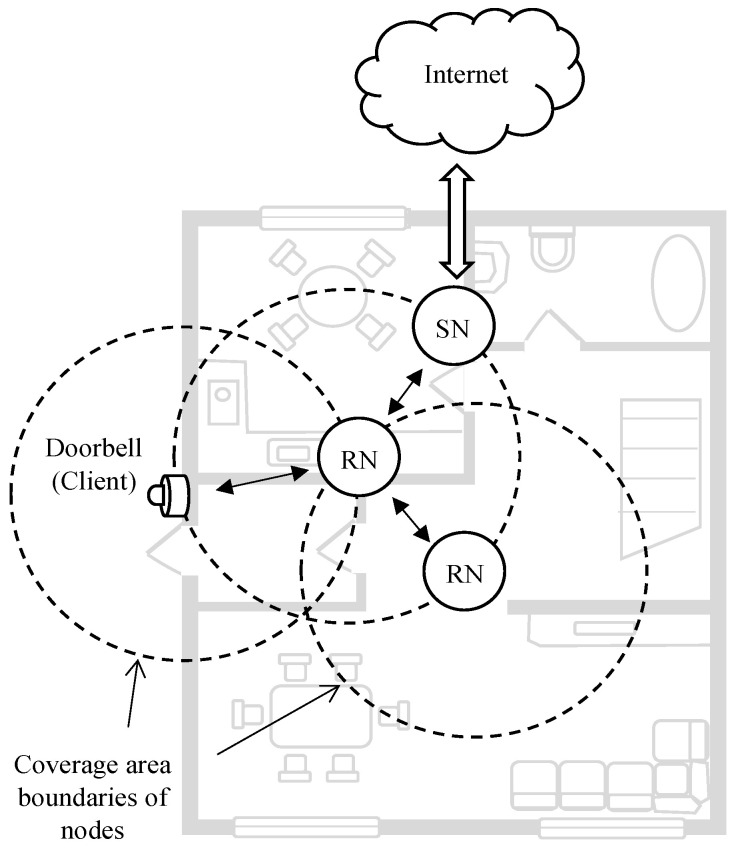
The BT Mesh network based smart doorbell communication structure, where “N” and “SN” denote a node and a server node, respectively.

As another example from literature using BT Mesh for smart building functions, a smart doorbell system was designed in [15] using BT Mesh networks. In [15], a domestic BT Mesh application together with a customised hardware was developed for a doorbell. The proposed application was implemented using Bluetooth Mesh over the BLE stack. In the work proposed in [15], several nodes were connected using the BT Mesh network to transmit messages. These nodes were classified into three groups: client nodes, server nodes, and relay nodes. Client nodes contain a Bluetooth system-on-chip (SoC) module with a button. When a visitor pressed the bell, the node connected to the doorbell sent a message to the server node (SN) via relay nodes (RNs). The SN acted as a gateway and was connected to the Internet. Once the SN received the the message, it sent to every mobile device on the network that a visitor was at the door. These were the only power constrained nodes in the system. In these nodes, messages were published via user interaction (i.e., when a user presses the button). Server anodes were made out of Bluetooth SoCs connected to the Internet via Arduino. UART was used to create the connection between the Bluetooth SoC and the Arduino module. The relay nodes were used to extend the coverage areas. Figure 3 depicts the overall structure of the proposed system.

It was shown in [15] that the proposed mechanism exhibits lower package losses across typical indoor environments compared to outdoor environments. Further, the method in [15] draws lower power for its functions, as expected.

#### 3.1.2. Smart Lighting

**Figure 4:**
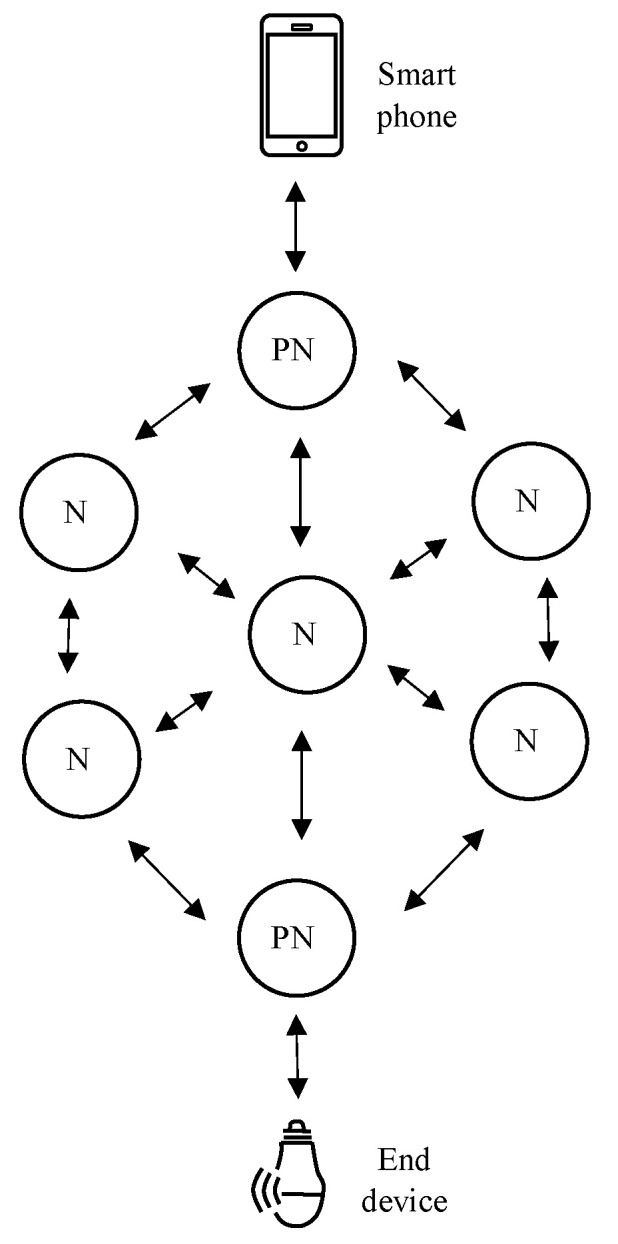
The concept of the data communication network presented in [21], where “N” and “PN” denote a node and a proxy node, respectively.

Wireless smart lighting is one of the key applications where BT Mesh has been predicted to be a disruptive technology. These systems can range from simple systems in smart homes to complex setups in commercial and industrial spaces, both indoors and outdoors. The potential of smart lighting systems have been discussed at length in the context of street lighting, warehouses, smart homes, and office spaces [34,35] (even though implementations employing BT Mesh are scarce). At the very basic level, a lighting system would have a group of lights that receives an ON/OFF signal, typically when a user flicks a switch (or a button in a smartphone app). Controlling the colour, brightness, and temperature of the lights, as well as sensor controlled functionality, add increased complexity. When the number of lights increases, the scalability and reliability of data transmission must be catered for. Lighting is inherently tied with human users, and therefore responsiveness in a way that feels natural is a critical requirement. In [21], authors proposed a mechanism to extend the control of light emitting diodes (LEDs) using a BT Mesh network. Figure 4 shows the proposed control system design. In this approach, a smartphone with BLE can be used to control an LED that is far away from the phone. In this mechanism, a smart phone will connect to the nearest bridging device and, via the device, which will be part of the BT Mesh network, a control signal will be sent to the end device. The experiments performed in [21] showed that the proposed mechanism can work well in an obstacle-free environment. The successful transfer rate of LED controlling signal at various distances was measured and the results were presented in [21]. From the results, it is clear that the transfer rate dramatically decreases after 24 m.

#### 3.1.3. Monitoring

BT Mesh has been employed to support the data transmission from sensors in [13,24,25,26]. In both [13,24], BT Mesh was used to transmit sensor data continuously. The experimental setup in [13] consisted of 33 nodes distributed in a 500 m${}^{2}$ office area, with all the sensors periodically generating data packets and propagating them towards a base station via a BT Mesh network. A similar setup was used in [24] as well. In both cases, the authors concluded that, although the systems were functional, BT Mesh is best suited for low data rate applications, such as a sporadic or event driven monitoring, and not suited for continuous monitoring applications where nodes have frequent/high data generation.

The aforementioned insights regarding low data rate applications were confirmed in the studies in [25,26], where data were not sent over the BT Mesh network continuously. In [26], if/when a hybrid node with a sensor and BLE beacon identifies a water leak, then the system starts to transmit leak notification messages via the BT Mesh towards an endpoint (e.g., a smartphone) at a maximum rate of once every four seconds. A similar setup seems to have been followed in the warning system using temperature and Hall effect current sensors in [25], although insufficient information is provided about the BT Mesh implementation. Both of the above projects report that the systems were operational and achieved their goals. However there is no evaluation of the BT Mesh performance.

#### 3.1.4. Disaster Communication

Having a communication system that can work after a natural or a man-made disaster (such as an earthquake) is an important and challenging task. This is because a disaster can cause physical damage to the infrastructure and, therefore, power and Internet cables may get damaged. As a result, communication systems that use either the Internet or the main power supply cannot function. To address this vital challenge, in [27], authors developed a BT Mesh network based system. The BT Mesh network is ideally suited for emergency communication (referred to as “Bluemergency”) for two main reasons. First, BT Mesh network facilitates many-to-many communication and therefore provides immunity against single-point-of-failure. Second, most of the BT Mesh devices can be made battery powered, thus they can withstand the main power outage. The solution proposed in [27] uses mesh devices together with mobile devices. The basic concept behind the system is that a mesh network that connects the nodes and mobile devices such as phones will communicate even when there is no main power supply and no Internet. A proof-of-concept implementation was performed using a test bed consisting of RuuviTags [36] sensors, Nordic Semiconductor nRF52840 USB Dongles [37], Raspberry Pi 3 nodes based on the Broadcom BCM2837B0 SoC, and smartphones Nexus 6P running Android version 8.1.0. The implementation in [27] was evaluated in terms of response time and packet-loss-rate. It was shown in [27] that the response time and packet-loss-rate were relatively lower. The biggest challenge in using the proposed method in [27] is that, generally, the system needs to operate where Wi-Fi signal interference may be higher.

#### 3.1.5. Smart Factory

For a smooth operation of a smart factory, it is very important to have a reliable, efficient, and secure communication system, as a smart factory is a highly digitized and connected production facility. The primary goal of the work presented in [28] was to develop a mechanism that can provide efficient, reliable, and secure communication in a smart factory environment. The main functionalities of the proposed work were to have a mesh sensor network to collect the data, facilitate downstream communication between IoT devices to the sensors, and assist secure communication that can guarantee data authenticity and integrity. A small scaled version of the proposed concept in [28] was implemented using Raspberry Pis with nRF52840 DK, which were powered by ARM Cortex-M4F CPUs.

#### 3.1.6. Smart Parking

**Figure 5:**
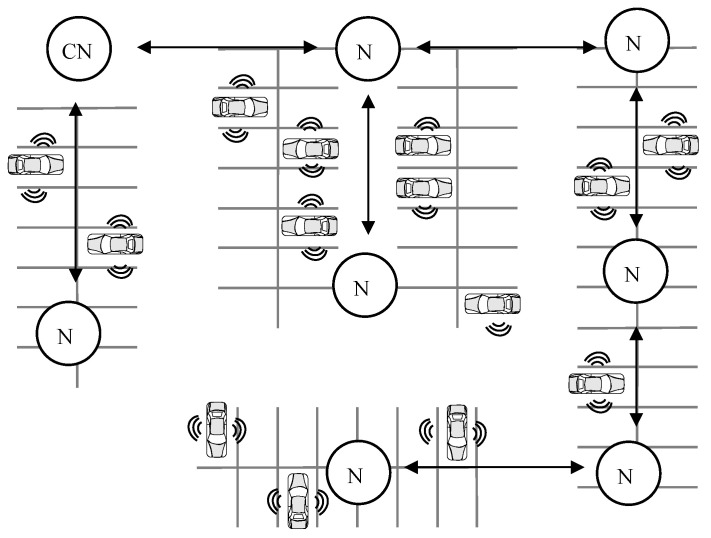
The overall structure of the smart parking system proposed in [30], where “N” and “CN” denote a node and a central node, respectively.

A BT Mesh network based smart parking system was developed in [30]. The primary motive behind the proposed work was to enhance the utilization of parking lots without using expensive technologies that require more power. The key component of a smart parking system is to easily and accurately identify the available parking lots. Authenticated nodes, referred to as sensor platforms, were placed in the parking lots. These nodes listened for broadcasts from a custom BLE beacon found in each parking vehicle in the lot. The nearby nodes gathered the received signal strength indication (RSSI) values from broadcasts. For the purpose of security, these RSSI values were encrypted and sent to their corresponding central node. By analyzing these RSSI values, the central node performed the prediction. The central node used an optimized random forest machine learning algorithm for prediction. In addition to the RSSI values, the machine learning algorithm also used features such as the average and median of RSSI values for a given time interval. In this work, BT Mesh network not only provided a large coverage area but also made the system robust against single-point-of-failure. The proposed system is depicted in Figure 5.

It was shown in [30] that the low power and low cost localization system proposed to identify the parking lot occupancy achieved detection accuracy of approximately 70%.

The findings of the above selected work are summarised in Table 2. Note that from the selected work, only those with experimental evaluation data (such as setup, metrics) are included in Table 2.

### 3.2. How BT Mesh Can Support IoT Application Requirements

**Figure 6:**
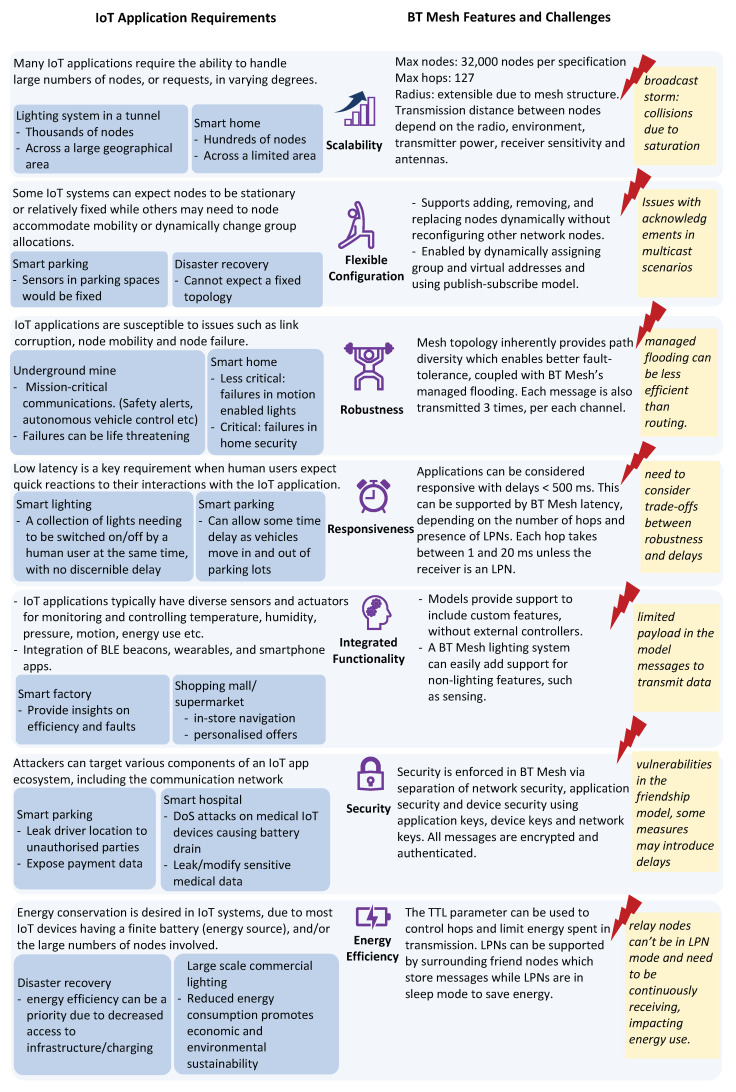
IoT application requirements mapped with BT Mesh features and challenges.

This section explores the feasibility of utilising BT Mesh in IoT applications considering seven requirement areas. As shown in Figure 6, the seven key IoT requirements of scalability, flexible configuration, robustness, responsiveness, integrated functionality, security, and energy efficiency are mapped with BT Mesh capabilities, as described below.

1. *Scalability*: Scalability refers to a system’s ability to handle growing amounts of work. IoT systems will need to be scalable in varying degrees, depending on the application context. For example, a wireless lighting system in an underground tunnel will need to cater to thousands of nodes, spread across a large geographical area, whereas the IoT devices in a smart home system will most likely consist of hundreds of nodes situated within a comparably smaller geographical area. BT Mesh can be used to cater to both extremes, due to the large number of nodes it can accommodate (32,000 nodes and 127 hops per packet at maximum, according to specs, although this will be lower in practice). Due to its mesh topology, BT Mesh can also cover an extended area. However, BT Mesh’s mechanism of controlled-flooding can cause the “broadcast storm” issue [6], where increased node saturation amounts to increased packet collisions, which poses a challenge for scaling up [24].
2. *Flexible Configuration*: For some IoT systems, it is essential to be able to support node configurations on the fly. There could be requirements to dynamically move nodes or to allocate nodes to different groups. Due to the mesh structure, BT Mesh networks can self-form and self-heal, which also provides fault-tolerance. BT Mesh standard supports dynamic assignment of the group and virtual addresses so that the physical network structure can be dynamically updated [7]. However, this method of dynamic group allocation using the publish–subscribe model also presents bottlenecks when a large number of nodes need to send acknowledgements of messages received to a single group owner [38]. For example, consider an IoT application for an underground mine where a controller node needs to send a mission-critical message (such as a notification to vacate an area due to a flammable gas leak) to hundreds of nodes. In this context, it is critical for the group owner to know whether the message has been received. However, because all of the hundreds of nodes in the group would be attempting to send their acknowledgements to the group owner at the same time, the network must manage high traffic, and there is a high probability of packet losses. A solution was experimentally implemented by Pierleoni et al. [38], where the acknowledgements are sent with a random delay to avoid simultaneous transmission, as suggested in the BT Mesh spec [39].
3. *Robustness*: Kitchenham et al. [40] defines robustness as “the degree to which a system or component can function correctly in the presence of invalid inputs or stressful environmental conditions”. In the context of IoT systems, considering the many heterogeneous devices interconnected together via various connection mediums, there is a high possibility of system errors occurring [41]. In some cases, failures in the system can be critical and even life-threatening, such as in autonomous vehicle control. BT Mesh provides fault-tolerance in the network due to its mesh topology that enables path diversity and its managed flooding [5]. However, although more fault-tolerant, the redundant transmissions mean that it is less efficient than single-path routing [5]. It is up to the developers to configure the degree of flooding according to the application context.
4. *Responsiveness*: IoT apps that involve human interactions require low latency so that the system can provide a quick reaction to fulfill human expectations. In particular, IETF RFC 5826 specifies interaction as real-time when the latency is below 500 ms [42]. BT Mesh can support this requirement, depending on the number of hops to reach the destination and if the nodes involve LPNs. Typically, in BT Mesh, each hop can take between 1 ms and 20 ms [5], unless the destination nodes are LPNs, in which case there can be additional delays, as LPNs can only receive data after polling its friend node/s. Developers can control latency by adjusting the BT Mesh parameters of advertising events and scanning events timing [6]; however, this needs to be balanced with implications for network robustness.
5. *Integrated Functionality*: IoT applications typically integrate multiple sensing, processing, connectivity, and actuating elements [43,44]. For example, integration of BLE beacons, mobile apps, wearables, and other IoT devices can provide localization, proximity detection, and activity sensing capabilities in numerous application areas, such as marketing, health monitoring, museum guiding, smart homes and offices, and warehouses [45]. Hence, it is particularly advantageous to use BT Mesh, which allows integrated functionality via its Models feature, which makes it easier to include custom features. For example, a BT Mesh lighting system can be embedded with other features, such as wireless lights also functioning as BLE beacons, or integrated with occupancy sensors [7]. A challenge is a limited payload available in messages used in BT Mesh models, which limits communication throughput. However, this may be alleviated to a considerable amount using pointers to other packets, i.e., packet chains [4].
6. *Security*: IoT architecture consists of components at the physical device layer, communication layer, and interfaces/services layer. This exposes IoT applications to attackers taking advantage of vulnerabilities in all these layers [46,47]. When considering security at the communication layer, BT Mesh has been designed with security as a first class citizen. Nodes in BT Mesh are provisioned using 256-bit elliptic curves and out-of-band authentication. To encrypt and authenticate the messages themselves, AES-CCM with 128-bit keys are used. Mesh operations and communications are secured via Network Key (to secure messages in nodes in the same network), Device Key (unique to each node, used to encrypt configuration messages), and Application Key (to encrypt and decrypt application data) [5,48]. According to the BT Mesh standard [39] the aforementioned security measures can provide protection against most threats against mesh networks. However, there are still vulnerabilities; device and application keys can be stolen/recovered by hardware exploitation and be susceptible to attacks such as simple power analysis, differential power analysis, and fault attack [49]. Other possible threats include malformation of the TTL value of packets [50] and exploiting “friend” nodes that lead to denial-of-service and impersonation attacks [51].
7. *Energy Efficiency*: Due to the limited battery power of hardware elements and the large number of devices involved in some cases, the need to conserve energy is important for IoT systems [52]. BT Mesh inherently supports this requirement via its BLE technology. The Upper Transport layer in BT Mesh protocol stack (See Figure 2) provides support for energy-constrained LPNs (see Section 2.1.2). LPNs are able to operate at reduced duty cycles with minimized usage of radio, with the help of nearby “friend nodes” (see Section 2.1.3). In addition, users can configure the TTLs to control the maximum hops per message, which acts as another energy conservation measure by ensuring messages are not relayed further than required [5,7]. Although flooding can be controlled via the TTL values, it is still more energy draining than routing, and the developers need to fine-tune the number of relay nodes and transmissions in a BT Mesh network to avoid unnecessary energy use. Moreover, LPNs are unable to act as relay nodes because relay nodes need to be in continuous scanning mode [4]. Hence, perhaps unsurprisingly, an experimental comparison of BT Mesh with 6BLEMesh has found 6BLEMesh to be more energy efficient for a given latency target [5] (see Section 5.1). To alleviate some of the aforementioned energy issues of flooding, the work in [53] proposed a simplified ContikiMAC mechanism [54], which lowers the receiving duty cycle, and by limiting the number of forwarded packets. In another study [55], the authors presented a strategy to reduce energy consumption in the friendship mechanism with burst transmissions and listen before transmit (BTLBT), showing a 19.81% improvement in a lifetime.

## 4. Comparing Bluetooth Mesh with Other Related Protocols

There are several wireless connectivity technologies available for IoT applications, including Wi-Fi, Zigbee (and other IEEE 802.15.4-Based Technologies), Z-Wave, cellular low-power wide area network technologies (NB-IoT, LTE-M), non-cellular low-power wide area network technologies (such as LoRaWAN, Sigfox), and conventional Bluetooth Piconet. In this section, we highlight the desirable features of BT Mesh compared to other technologies.

Modern industries require highly reliable, scalable, secure, low-cost, and low-powered wireless technologies for their smooth and efficient functioning. BT Mesh networks satisfy all the aforementioned requirements compared to other methods. As BT Mesh supports many-to-many device communications, it is ideally suited for creating large-scale device networks.

Currently, BT Mesh is extensively used for smart lighting systems and sensor networks. As BT Mesh-based systems can facilitate the communication of thousands of devices, BT Mesh is highly welcomed in the IoT environment.

**Wi-Fi** is based on the IEEE 802.15.4 technical standard. Wi-Fi can provide bandwidth up to 2 MHz. As a result, Wi-Fi communication networks are widely used applications where higher-bandwidth data transfers are required (e.g., transferring large multimedia files). They cannot be used in battery-powered systems as Wi-Fi consumes relatively higher power.

**Zigbee** technology was developed based on the IEEE 802.15.4 technical standard. It is popular for its low power consumption and low data rates. Unlike Wi-Fi, Zigbee does not require much power. It is primarily developed to carry small amounts of data over short distances. Zigbee is not well suited for applications involving larger IoT-based systems or networks. Further, Zigbee can only be used for short-range connectivity.

**Z-Wave** technology was originally developed for home automation. Z-Wave devices use the radio frequency of 908.42 MHz, unlike the congested 2.4 GHz spectrum used by Wi-Fi and Zigbee. Due to this, Z-Wave devices have lower levels of interference compared to Wi-Fi and Zigbee based devices. Because Z-Wave technology is customised for home automation, it supports a large number of devices. In most cases, similar to Zigbee, Z-Wave also has a singular point of failure. Due to this, Z-Wave cannot be used in IoT applications that require high reliability.

**NB-IoT** and **LTE-M** were both developed for IoT applications by 3rd Generation Partnership Project (3GPP) (https://www.3gpp.org/about-us accessed on 15 January 2022). These two technologies are vital parts of long-term 5G IoT technology. NB-IoT is mainly used in applications where low-power and low-bandwidth devices can be used. In contrast, LTE-M provides a higher data rate. Hence, LTE-M is used in time-demanding applications. As both NB-IoT and LTE-M primarily use star topology, they are vulnerable to single-point failure.

**LoRaWAN** and **Sigfox** are both non-cellular low-power wide area network technologies, popularly used in smart city applications such as smart electricity meters and smart parking. LoRaWAN is an open-source wireless networking protocol built on top of a proprietary modulation format called “LoRa”. LoRaWAN is popular in the US. The proprietary technology Sigfox is a subset of LoRaWAN technologies that is popular in EU regions. Both LoRaWAN and Sigfox use star topology and are vulnerable to single-point failure.

**BT Piconet** is an adhoc network that connect devices via BT technology. Typically, Piconet connects a wireless user group of devices using Bluetooth technology protocols. In this technology, one device needs to act as master, and it can be connected to a maximum of seven active slave devices at a given time. The master device can be connected to 255 inactive slave devices. The master device can make an inactive device active when needed. As the master device can be connected to only seven active slave devices, it limits the network and data sharing capability of Piconet.

Table 3 compares BT Mesh technology to Wi-Fi, Zigbee, Z-Wave, NB-IoT, LTE-M, LoRaWAN, Sigfox. and BT Piconet technologies in terms of range, throughput, power consumption, ongoing cost, and topology.

From our analysis, we found that different networking technologies have different characteristics and are therefore suitable for different types of applications. For example, Wi-Fi is preferred where high data transmission rates are needed. However, because Wi-Fi consumes more power, it cannot be deployed in power constrained applications. Similarly, if we consider BT Piconet, it can be very useful in personal area networks where typically a limited number of devices need to communicate over a short distance. However, BT Piconet cannot be effectively used in scenarios where a large number of devices need to interact over a bigger area. When it comes to BT Mesh, it has some unique advantages that can be summarised as follows:

- As BT Mesh does not use a central hub for communication and uses a mesh networking, the connectivity is more reliable and can avoid single-point failure.
- BT Mesh technology can be easily extended to cover a larger area. This is possible as there is no single master device used in this technology such as the one used in BT Piconet.
- The low power consumption of BT Mesh makes it ideally suited for power demanding applications where a main power supply is not available.
- Due to the interoperability of BT Mesh between different manufacturers, it can be used in diverse contexts.
- BT Mesh is scalable and, as a result, it can be modified easily even after it is implemented.
- BT Mesh nodes are secure, as the system provides end to end security for messages exchange between devices.

Although BT Mesh has a number of advantages, it cannot be used applications such as high-quality video streaming, where high data transmission rate is required. Unlike Wi-Fi technology, BT Mesh cannot achieve a data transfer rate of more than 2 Mbps.

## 5. Experiments in Related Work

As BT Mesh is an emerging and evolving technology, only a very limited number of experiments have been performed in the literature.

### 5.1. BT Mesh versus 6BLEMesh

In order to satisfy the market demand, two different Bluetooth Mesh-based technologies were developed, BT Mesh and IPv6-based BLE mesh networks (6BLEMesh), by Bluetooth SIG and the Internet Engineering Task Force (IETF), respectively [5]. In [5] authors compare these two technologies mainly in terms of protocol encapsulation overhead, latency, energy consumption, message transmission count, and corruption robustness, as follows:

- *Protocol encapsulation:* In [5], protocol encapsulation is defined as “total header and footer overhead added by all protocol stack layers to a user data payload before transmission”. From the data reported in [5], we can see that both BT Mesh and 6BLEMesh have nearly similar overheads.
- *Latency:* To asses the latency, delays in packet transmission over multihop paths were measured. It was presented in [5] that in BT Mesh each hop contributes at least the time required to transmit a packet via the advertising channels. In 6BLEMesh, the time necessary to transfer a packet from one node to its next node is a uniformly distributed random variable.
- *Energy consumption:* In [5], current consumption is measured based on an nRF51 DK hardware platform and a battery capacity of 235 mA. As expected, for both BT Mesh and 6BLEMesh, the battery lifetimes were primarily based on the parameter values chosen. Form the data presented in [5], we can conclude that for a given latency goal, devices consume less energy in 6BLEMesh compared to BT Mesh.
- *Message transmission count:* From different network sizes, node densities, and protocol parameters, the total number of message transmissions are counted. One can see from the data provided in [5] that BT Mesh exhibited a higher number of data message transmissions compared to 6BLEMesh. This is due to the fact that BT Mesh and 6BLEMesh use managed-flooding and single-path routing, respectively.
- *Link corruption robustness:* Robustness of both technologies against radio signal fading or interference was assessed in [5]. Robustness was measured in terms of end-to-end packet delivery probability. It can be observed from [5] that BT Mesh achieved a higher packet delivery rate when there was diversity in the paths. On the other hand, 6BLEMesh showed excellent packet delivery performance when the maximum number of consecutive link-layer retries in a Link layer connection was set to a sufficiently large value.

It should be noted that the implementation level details of the experiments/simulations were not provided in [5].

### 5.2. Reliability, Delay, and Scalability Analysis

**Figure 7:**
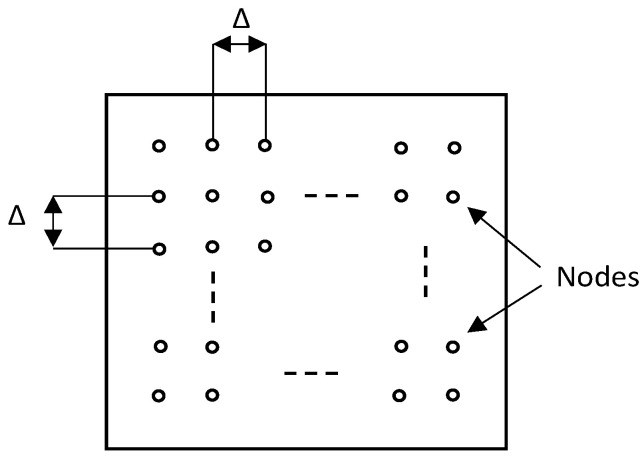
The structure of the network topology used in [6].

In [6], authors evaluated the performance of BT Mesh in terms of reliability, delay, and scalability using simulations. For the evaluation, grid topology was used where all devices take the role of relay nodes. The separation between two nodes is denoted by Δ, as shown in Figure 7. Four different versions of the topology were used by varying the separation Δ and number of devices.

The radio range was assumed to be 9 m, and the maximum PDU length was set to 39 octets. Experiments were performed using a simulation tool developed using MATLAB. To simulate the interference, WLAN interference data gathered from an indoor office environment were used.

In order to evaluate the reliability, for different time intervals between consecutive transmissions within an Advertising Event ($T_{iPDU}$s), congestion probabilities were compared against packet error rates (PERs). From the data presented in [6] it can be seen that congestion probability was significantly lower when shorter $T_{iPDU}$s were used across different PERs. It clearly shows that shorter $T_{iPDU}$s can maximize reliability.

From the simulations, it was reported in [6] that when the number of available paths was fewer and the lengths were longer, delay increased, as expected. We can see from the data presented in [6] that the average, as well as the maximum delays, were increased when larger $T_{iPDU}$s were used compared to smaller $T_{iPDU}$s.

Based on the simulations, in [6], authors predicted that the scalability of BT Mesh network can be increased by improving the channel usage, properly designing channel hopping patterns, and outlining appropriate policies for the spatial distribution of relay nodes.

### 5.3. Evaluation of a Framework for Collecting Network Traffic Data

In [8], Karlson presented a framework for collecting network traffic data that can be utilized to assess the performance of BT Mesh networks. In [8], implementation details of the framework, based on the BT Mesh network are provided.

The performance of the framework was evaluated primarily in terms of memory utilization, effects on user traffic, and latency.

- *Memory utilization:* The additional memory used on nodes was measured when the framework was deployed compared to nodes without the framework. From the data presented in [8], we can see that more memory was used when the framework was deployed, as expected. From the information provided in [8], in terms of percentage, we can say that the extra memory usage was small. The analysis presented in [8] only took static use of the available memory and it did not take into account the stack and the heap (which are also stored in RAM).
- *Effects on user traffic:* To measure the network traffic, the packet delivery ratio (PDR) was used as the metric. From the experimental data in [8], one can see that PDR was reduced with the distance between transmissions. This can be expected as with increasing distance, the probability of packets colliding and interfering with each other reduces.
  A greater distance between transmissions reduced the probability of packets colliding and interfering with each other and, therefore, decreased the PDR. Further, from the experiment outcomes, we can observe that PDRs decreased with increasing message sizes.
- *Latency:* The experiments were primarily designed to measure the transmission delay against message sizes. From the presented data in [8], one can clearly see that delay linearly increased with the message size, as anticipated. From the experimental results, it is evident that the relaying process introduced uncertainty in the delay. This can be expected as relaying generates more traffic, which will increase the probability of collision.

### 5.4. Evaluation of the BT Mesh Protocol for Monitoring Applications

In [56], in the context of monitoring applications, authors evaluated the performance and maximum throughput of the BT Mesh protocol under multi-hop implementation.

For the experimentation, 33 nodes were placed in an indoor office environment. There were obstacles such as desks, monitors, and other electronic devices in the environment, which mostly did not allow clear line-of-sight between nodes. Further, there were some Wifi hot-spots present in the area. These Wifi hot-spots created a noisy radio frequency environment. In [56], four important experiments were conducted as follows:

- *Full-scale one-hop:* The goal of this experiment was to assess the full potential of the BT Mesh network under different traffic conditions. In this experiment, relay nodes were not used. To evaluate the performance of the BT Mesh under a radio-frequency signal environment, experiments were conducted both during work hours and during off-hours. The PDR was primarily used to measure performance. From the experimental results, it can be seen that there is an approx. 10% reduction in overall packet reception ratio due to the interference from other radio technologies such as Wifi.
- *Small-scale multi-hop:* The primary aim of this experiment was to evaluate the processing and transmitting packet capacity of the relay nodes. From the experiments, authors calculated the maximum amount of packets relays can process. It can be noted from the experiments that relays became unreliable when they were overloaded.
- *Full-scale multi-hop:* In this experiment, the overall performance of the network was tested in multi-hop communication scenarios with an increasing number of nodes. From the experimental results, one can see that the PDRs significantly varied based on the location of the nodes. This is mainly caused by the inability of the relay nodes to process a larger amount of packets per second.
- *Nomadic Node Experiments:* In [56], patients with wireless sensors were referred to as nomadic nodes. These nodes were expected to move in the environment. For convenience, all nomadic nodes were attached to the same individual. The parameters are adjusted to make the nomadic node functional by reducing the stress on the relay nodes. However, adequate details of the experimentation results using nomadic Node were not provided in [56].

### 5.5. Performance Assessment of BT Mesh in Terms of Latency, Round Time Trip (RTT) Delays, and Energy Consumption

In [57], the authors performed experiments to test the performance of BT Mesh using the Nordic nRF51 Development Kit and the Nordic nRF51 Dongle in the implementation.

- *Latency and RTT experiment:* In this part of the experiment, several tests were conducted in [57]. Overall, the results show that the RTT increased with an increasing number of hops, as expected. Furthermore, it can be seen from the results presented in [57] that RTT increased in a non-linear manner with the number of nodes.
- *Energy consumption:* In this experiment, current consumption and battery lifetime of the devices were tested. The goal of the experiment was to generate a current consumption model for low-power devices. Details of the experimental setups are provided together with results in [57]. From the results, we can see that, compared to the normal expectancy of 10 years, the experiments and the related calculations show a lifetime of slightly less than 2 years. It should be noted that in [57], authors considered sleep-mode current consumption in the devices used. It can also be observed in [57] that there is a sensitivity of battery lifetime to parameters such as receive window size.

### 5.6. Evaluating the Latency Performance of BT Mesh

In [1], authors evaluated the performance of the BT Mesh in terms of latency using Nordic nRF52832 modules to conduct experiments. Experiments were performed with an increasing number of nodes, starting with two nodes. The following three types of measurements are made in [1]:

- *Baseline Measurement:* To have a reference level RTT that was measured with just two nodes;
- *Multiple Neighbors Measurement:* Measurements are made with more neighbours. As expected, RTT reduced with more neighbours;
- *Multiple Hops Measurement:* In this measurement, communication flows that span multiple hops in a BT Mesh were considered a network. It can be seen from the data that RTT almost linearly increased with a number of hops.

Further, in [1], the effect of external BLE interference was tested. Understandably, RTT increased with more external BLE beacons.

### 5.7. Energy Consumption with Flooding Protocols

The energy consumption of nodes determines the battery life. Therefore, it is important to increase battery lifetime by reducing energy consumption. In [58], authors proposed an energy-balanced flooding algorithm called “Drypp” and compared it with the popular flooding algorithm Trickle. For the experiments, several hardware modules, tools, and simulators, including Nordic Semiconductors Software Development Kit (SDK), FruityMesh framework, and RedBear R Nano V2 development boards with the Nordic R nRF52832 micro-controller, were used. Details of all the modules used in the experiments are presented in [58] with references. The following two scenarios were used in the comparison of Trickle and Drypp flooding protocols:

- *Tandem scenario:* In this scenario, authors created a maximum range topology where nodes were separated by 1.5 m. The network was designed in such a way that all the nodes needed to be active to make the network functional. From the data presented in [58] we can see that the throughput of Tickle was comparable to Drypp. Further, the Tickle protocol had a higher percentage of packet transmissions than the Drypp protocol.
- *Parallel scenario:* In this scenario, the performance of Tickle and Drypp were compared when they were applied in a parallel topology [58]. The data clearly show that the throughput of Drypp and Trickle were converging with time and were relatively closer to each other. In the context of battery by time, the difference between the fall time of each node changed in a divergent manner.

Table 4 summarises findings from experiments on BT Mesh found in related work that was detailed in this section. The Xs in the table denote aspects that were investigated via experiments or simulations. It should be noted that we have only included work that provided adequate details about the experiments, such as device setups and test results. Although [5,58] are discussed in the section, they are omitted from Table 4, as their main focus was to compare BT Mesh versus 6BLEMesh, and to evaluate a flooding algorithm, respectively, whereas the table focuses on work that evaluates the performance of BT Mesh.

As can be seen in Table 4, aspects related to transmission delays/latency/RTT were the most investigated, whreas not many experimental data exist for scalability, mobility, and energy consumption. None of the reviewed existing related work provided data on experiments regarding BT Mesh groups at all.

## 6. Experiments in Group Messages

Although some related work has mentioned BT Mesh’s group messaging capability, there are very few data and little analysis from actual experimental investigations (see Section 5). Hence the group messaging behaviour in BT Mesh networks and its implications for scalability and reliability are not very well understood. To investigate this, in this section, we present our findings from a set of experiments examining the effect of managed flooding in group messaging in BT Mesh.

### 6.1. Experiment Setup

Ten BT Mesh nodes and one gateway device were used in this experiment. The nodes were provisioned into the Bluetooth Mesh network and added to the same group. Three different tests with varying node proximity were conducted. The initial TTL value was set at 4. In each test, LED ON/OFF group messages were sent to the network, and time and debug messages were recorded through the host computer via the gateway device. The sent time in the group message and acknowledgement received time in each node were recorded. This was repeated ten times for each test. The LED ON/OFF state on each node was also visually observed to make sure that the group message was received by the node.

- Test 1: The nodes and the gateway device were set up in close proximity ( 1–5 cm between each other), and group messages were sent to the nodes.
- Test 2: The nodes and the gateway device were set up with approximately 10 m distance between each node and the gateway (in a star topology), and group messages were sent to the nodes.
- Test 3: The nodes and the gateway device were set up approximately 10 m between nodes and the gateway in a linear arrangement, and group messages were sent to the nodes.

**Figure 8:**
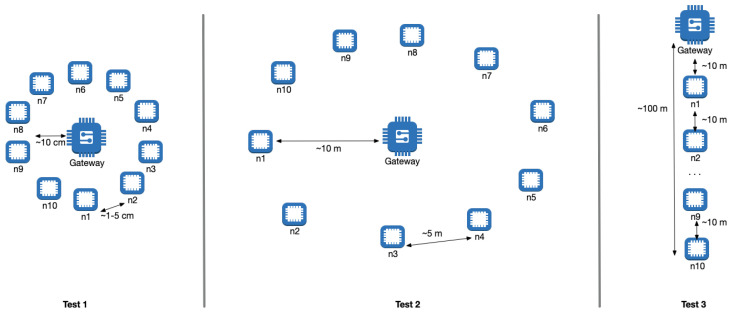
Setup of group messaging experiments.

Figure 8 shows the topologies of the three tests.

### 6.2. Experimental Results

We examined the TTL values in the experiment logs for each message transfer in each node to find out the transmission path. In all tests, the TTL values varied between 2 and 4, which reflects the number of hops in the transmission path. In most cases, the messages were sent directly to the gateway. However, surprisingly, even in Test 1, when each node was at most 5 cm from the gateway, some acknowledgment messages only reached the gateway via other nodes, hence taking a longer path. This pattern was prevalent throughout the three tests (ranging from 5% to 11%). Another interesting observation was the amount of dropped acknowledgments. In Test 1, the gateway received 91% of the acknowledgments from the group nodes, even though it was observed that the message was received by the node (by visually verifying the LED state). This value dropped to 78% in Test 2 and 63% in Test 3, even though the messages were received by the nodes.

### 6.3. Discussion

The cause of messages taking a longer path can be explained by BT Mesh’s managed-flooding, which causes messages to follow multiple paths, rather than a set route (see Section 2). This behaviour is “managed” by the heartbeat and TTL mechanisms. The impact of taking a longer path depends on how time-sensitive the system is. Although the effect of this behaviour in the experiment with 10 nodes was negligible, it will be interesting to investigate the impact at a larger scale. The case of missing acknowledgments corroborates another experimental study by Pierleoni et al. [38]. In fact, the root cause of this behaviour lies in the BLE simultaneous advertising when multiple nodes send data to the same receiver at the same time [59]. For example, if two nodes transmit two messages roughly simultaneously (where the time difference between the two transmission events is within 0.1 s–1 s) there is a potential for packet collision, leading to the loss of the message. As evident by our test results, the acknowledgment packet loss seems to be further amplified by the distance between nodes. This behaviour can be a serious concern in applications where it is critical to know whether a message has been received (see Section 3.2).

## 7. Challenges Related to Bluetooth Mesh

In this section, we discuss some of the major challenges faced by BT Mesh together with the limitations.

- BT Mesh networks experience interference due to the presence of other signals such as Wi-Fi signals. Randomization of the timing parameters could alleviate some of the effects of this interference [60].
- Nodes such as relays/friends are expected to scan the network continuously. However, due to the limitations in firmware/hardware, scanning procedures do not occur continuously. As a result, the packets received within the non-scanning period are lost. However, in general, except in the case of acknowledgment messages, this is not a significant loss due to BT Mesh’s controlled flooding and redundant transmissions where each message is transmitted three times [5].
- BT Mesh networks do not support streaming high data rate applications such as audio streaming. For example, using BT Mesh networks, one cannot stream music to all the speakers in their home. This is because BT Mesh networks use BLE that is not designed for continuous wireless connections. As technology improves, including increased bandwidth in future BT Mesh versions, a wider range of software or firmware profiles might support a larger range of applications, including multimedia.

## 8. Conclusions and Future Directions

BT Mesh is an emerging technology that can support IoT environments with reliable communications. This paper provides an overview of BT Mesh that includes a brief description of the elements in BT Mesh protocol stack and details about different node types. The real-life implementation of BT Mesh are also presented with its performance. Further, the suitability of BT Mesh to IoT applications is discussed in terms of scalability, flexibility, robustness, responsiveness, integrated functionality, security, and energy efficiency. To give the readers the pros and cons of BT Mesh over other communication technologies, we have provided a comparison of BT Mesh with technologies such as Wi-Fi, Z-Wave, Zigbee, and LTE-M. To find the effect of managed flooding in group messaging in BT Mesh, we performed experiments and reported our findings. In addition, this paper also discusses current challenges related to BT Mesh with some avenues for addressing them. Analysis of current state-of-the-art applications implemented using BT Mesh demonstrate the applicability of BT Mesh technology in many types of IoT applications in various domains. However, much work still remains.

BT Mesh uses several performance influencing parameters/functions such as time-to-live (TTL) and relay features. The feature configuration and parameter selection can have a huge impact on the performance of the BT Mesh network, such as a reduction in reliability and higher energy consumption. Further work is required to experimentally understand the effect of different parameter settings on performance, reliability, and energy consumption in various settings.

BT Mesh is useful for a collection of nodes within close range to inter-connect or to perform proximity sensing and localization for limited bandwidth operations; further studies on how varying mobility situations (e.g., speeds and types of mobility) might affect performance and robustness in harsh environments are still needed, e.g., as our experiments show that a node receives some of its messages from other nodes instead of directly from the sender node suggests a robustness mechanism, so that if multiple copies of a message are sent through the mesh network and some nodes move out of immediate range of a node, a node can still receive the message from remaining in-range nodes.

A wider range of applications together with best practices for BT Mesh can be explored even as the technology is deployed in more applications; this can lead to, first, best practices and parameter settings for the deployment of a BT Mesh for a certain environment and application domain to achieve best performance (including, for example, how many nodes for coverage of a certain area with particular features with redundancy for robustness, the topology in which the nodes should be connected, the transmission properties for maximizing the lifetime of nodes or the network, the configuration for minimizing energy use, and so on), and second, to subsequent recommendations for future BT Mesh developments.

BT Mesh embedding is another interesting direction, where nodes are embedded into everyday things and into common structures (e.g., within walls of a place or in a construction site), lighting systems in a building to street furniture and (instrumented) everyday objects and appliances in the home for smart home applications, or in factories and commercial places (e.g., health monitoring or aged care); such embedding can allow “ubiquitous connectivity” among everyday things but will need to be studied with trade-offs in maintenance over long periods, operational costs, and benefits.

## Figures and Tables

**Table 1:** Classification of IoT applications using Bluetooth Mesh in related work.

Category	Use of BT Mesh
Smart Buildings	BT Mesh has been used to support a variety of features in smart buildings, including smart homes and commercial/office buildings. These include indoor localisation features [12,13,14], smart doorbells [15], and other smart home functions [16,17,18,19,20].
Smart Lighting	Applications for wireless smart lighting can include various indoor and outdoor settings. Scenarios explored in the literature include smart homes [21], smart office spaces [22], and smart city traffic lights [23].
Monitoring	Monitoring systems can include observing human behaviour and environmental conditions as well as keeping track of the health of critical equipment via sensors. Related work has investigated the feasibility of using BT Mesh to support such monitoring scenarios in [13,24,25,26] experimentally, emulating office and lab settings, where BT Mesh was used to deliver the sensor data packets.
Disaster Communication	BT Mesh based communication systems can be an alternative in cases of lack of Internet connectivity when communication infrastructure breaks down in disaster scenarios such as proposed in [27].
Smart Factory	Industrial environments can use BT Mesh to support efficient human–machine and machine–machine communication as investigated in [28,29].
Smart Parking	BT Mesh can be employed to implement infrastructure-less ticketing and management of parking lots, as proposed in [30,31], aided by BLE beacons.

**Table 2:** Validated Bluetooth Mesh applications discussed in related work.

Goal	Examples from Literature	Metrics in Literature	Findings from Literature
Extend the range of control/messaging	Smart doorbell	Package losses	Proposed mechanism exhibits no lower package loss when the distance is less than or equal to 10 m; when the distance is less than or equal to 24 m, the packet loss is less ≤20%.
	LED control system extension	Transfer rate	Transfer rate dramatically decreases after 24 m
	Smart factory	Average time taken, average message interval, message loss, message data rates, round trip time	When 500 messages are used, min. average time taken = 6569 ms, min. average message interval = 13 ms, max.message data rate = 76 msg/s, min. round trip time 109 ms, and there are no messages lost.
Localisation using RSSI	Secure smart parking lot	Detection accuracy	Detection accuracy of approximately 70%
Enable communication in the absence of infrastructure	Post-disaster communication system	Response time, packet-loss-rate	In the smart office, response time (ms) and packet loss rate (%) are 1053.13 and 38.21, respectively. In the smart home, response time (ms) and packet loss rate (%) are 995.53 and 8.5, respectively.

**Table 3:** The comparison of BT Mesh with other technologies.

Attributes	Technologies								
	BT Mesh	Wi-Fi	Z-Wave	Zigbee	LTE-M	NB-IoT	Sigfox	LoRaWAN	BT Piconet
Range	100 m–1 km	15 m–100 m	30 m–50 m	30 m–100 m	1 km–10 km	1 km–10 km	3 km–50 km	2 km–20 km	<10 m
Through-put	125 kbps–2 Mbps	54 Mbps–1.3 Gbps	10 kbps–100 kbps	20 kbps–250 kbps	≤1 Mbps	≤200 kbps	≤100 bps	10 kbps–50 kbps	200–2100 kbps
Power consumption	Low	Medium	Low	Low	Medium	Low	Low	Low	Medium
Ongoing cost	One-time	One-time	One-time	One-time	Recurring	Recurring	Recurring	One-time	One-time
Topology	Mesh	Star, Mesh	Mesh	Mesh	Star	Star	Star	Star	Star

**Table 4:** BT Mesh evaluations in related work.

Related Work	[6]	[8]	[56]	[57]	[1]
**Type**	**Simulations**	**Experiments**	**Experiments**	**Experiments**	**Experiments**
Reliability	X	-	-	-	-
Delay/Latency/RTT	X	-	X	X	X
Scalability	X	-	-	-	-
PDR	-	X	X	-	-
Mobility	-	-	X	-	-
Energy consumption	-	-	-	X	-
Groups	-	-	-	-	-

## Data Availability

Not applicable.

## References

[1] Baert M., Rossey J., Shahid A., Hoebeke J. (2018). The bluetooth mesh standard: An overview and experimental evaluation. Sensors.

[2] Yin J., Yang Z., Cao H., Liu T., Zhou Z., Wu C. (2019). A survey on Bluetooth 5.0 and mesh: New milestones of IoT. ACM Trans. Sens. Netw. (TOSN).

[3] Ghori M.R., Wan T.C., Sodhy G.C. (2020). Bluetooth low energy mesh networks: Survey of communication and security protocols. Sensors.

[4] Hernandez-Solana A., Perez-Diaz-De-Cerio D., García-Lozano M., Bardaji A.V., Valenzuela J.L. (2020). Bluetooth Mesh Analysis, Issues, and Challenges. IEEE Access.

[5] Darroudi S.M., Gomez C., Crowcroft J. (2020). Bluetooth low energy mesh networks: A standards perspective. IEEE Commun. Mag..

[6] Rondón R., Mahmood A., Grimaldi S., Gidlund M. (2020). Understanding the performance of bluetooth mesh: Reliability, delay, and scalability analysis. IEEE Internet Things J..

[7] Woolley M., Schmidt S. (2017). Bluetooth Mesh Networking: Paving the Way for Smart Lighting. https://www.bluetooth.com/bluetooth-resources/bluetooth-mesh-paving-the-way-for-smart-lighting/.

[8] Karlsson S. (2019). A Data Collection Framework for Bluetooth Mesh Networks. Master’s Thesis.

[9] Xia N., Chen H.H., Yang C.S. (2019). Emerging technologies for machine-type communication networks. IEEE Netw..

[10] Tekler Z.D., Low R., Gunay B., Andersen R.K., Blessing L. (2020). A scalable Bluetooth Low Energy approach to identify occupancy patterns and profiles in office spaces. Build. Environ..

[11] Esrafilian-Najafabadi M., Haghighat F. (2021). Occupancy-based HVAC control systems in buildings: A state-of-the-art review. Build. Environ..

[12] Jürgens M., Meis D., Möllers D., Nolte F., Stork E., Vossen G., Werner C., Winkelmann H. Bluetooth Mesh Networks for Indoor Localization. Proceedings of the 2019 20th IEEE International Conference on Mobile Data Management (MDM).

[13] Montecchiari L., Trotta A., Bononi L., Di Felice M. Bluetooth Mesh Technology for the Joint Monitoring of Indoor Environments and Mobile Device Localization: A Performance Study. Proceedings of the 2022 IEEE 19th Annual Consumer Communications & Networking Conference (CCNC).

[14] Tong X., Wang L., Cui Y. Research on Indoor Positioning Based on Smart Home Bluetooth Networking. Proceedings of the 2022 International Conference on Artificial Intelligence and Computer Information Technology (AICIT).

[15] Martínez C., Eras L., Domínguez F. The Smart Doorbell: A proof-of-concept Implementation of a Bluetooth Mesh Network. Proceedings of the 2018 IEEE Third Ecuador Technical Chapters Meeting (ETCM).

[16] Taştan S.İ., Dalkiliç G. Smart Home System Using Internet of Things Devices and Mesh Topology. Proceedings of the 2021 6th International Conference on Computer Science and Engineering (UBMK).

[17] Tran Q.T., Tran D.D., Doan D., Nguyen M.S. An Approach of BLE Mesh Network For Smart Home Application. Proceedings of the 2020 International Conference on Advanced Computing and Applications (ACOMP).

[18] Dvoynikov V.M., Smirnov V.A., Burilov D.A. Comparative Analysis of Mesh and Thread Networks and their Application Possibility in the "Smart Home" Systems. Proceedings of the 2021 IEEE Conference of Russian Young Researchers in Electrical and Electronic Engineering (ElConRus).

[19] Sergi I., Montanaro T., Gammariello M.C., Patrono L. The use of Bluetooth Mesh Networking in IoT-aware Applications. Proceedings of the 2021 6th International Conference on Smart and Sustainable Technologies (SpliTech).

[20] Zheng X., Xue S., Cao H., Wang F., Zhang M. A cost-efficient smart IoT device controlling system based on bluetooth mesh and cloud computing. Proceedings of the 2020 Chinese Automation Congress (CAC).

[21] Lee T.Y., Truong P.H., Lee C.K., Jeong G.M. Range extension of LED control systems using a Bluetooth mesh network. Proceedings of the 2017 IEEE International Conference on Consumer Electronics (ICCE).

[22] Abboud K., Li Y., Bermudez S. (2020). eSNAP: Enabling Sensor Network Automatic Positioning in IoT Lighting Systems. IEEE Internet Things J..

[23] Veiga A.A., Abbas C.J. (2018). Proposal and application of Bluetooth mesh profile for smart cities’ services. Smart Cities.

[24] De Leon E., Nabi M. An experimental performance evaluation of bluetooth mesh technology for monitoring applications. Proceedings of the 2020 IEEE Wireless Communications and Networking Conference (WCNC).

[25] Dvoynikov V.M., Smirnov V.A., Burylov D.A. Implementation of a Monitoring System for an Electrical Network Based on a Contactless Temperature Sensor and a Hall Effect Current Sensor. Proceedings of the 2022 Conference of Russian Young Researchers in Electrical and Electronic Engineering (ElConRus).

[26] Witham O., Johnston L.N., Xiao M., Feng J., Zhou N., Shaker G. Batteryless wireless water leak detection system. Proceedings of the 2019 International Conference on Smart Applications, Communications and Networking (SmartNets).

[27] Álvarez F., Almon L., Radtki H., Hollick M. Bluemergency: Mediating Post-disaster Communication Systems using the Internet of Things and Bluetooth Mesh. Proceedings of the 2019 IEEE Global Humanitarian Technology Conference (GHTC).

[28] Yang Lam T.C., Ling Yew S.S., Keoh S.L. Bluetooth Mesh Networking: An Enabler of Smart Factory Connectivity and Management. Proceedings of the 2019 20th Asia-Pacific Network Operations and Management Symposium (APNOMS).

[29] Garrido-Hidalgo C., Hortelano D., Roda-Sanchez L., Olivares T., Ruiz M.C., Lopez V. (2018). IoT heterogeneous mesh network deployment for human-in-the-loop challenges towards a social and sustainable Industry 4.0. IEEE Access.

[30] Seymer P., Wijesekera D., Kan C. Secure Outdoor Smart Parking Using Dual Mode Bluetooth Mesh Networks. Proceedings of the 2019 IEEE 89th Vehicular Technology Conference (VTC2019-Spring).

[31] Seymer P., Wijesekera D., Kan C.D. (2021). Smart Parking Zones Using Meshed Bluetooth Sensor Networks. Proceedings of the International Conference on Smart Cities and Green ICT Systems, International Conference on Vehicle Technology and Intelligent Transport Systems.

[32] Al Dakheel J., Del Pero C., Aste N., Leonforte F. (2020). Smart buildings features and key performance indicators: A review. Sustain. Cities Soc..

[33] Akkaya K., Guvenc I., Aygun R., Pala N., Kadri A. IoT-based occupancy monitoring techniques for energy-efficient smart buildings. Proceedings of the 2015 IEEE Wireless communications and networking conference workshops (WCNCW).

[34] Füchtenhans M., Grosse E.H., Glock C.H. (2021). Smart lighting systems: State-of-the-art and potential applications in warehouse order picking. Int. J. Prod. Res..

[35] Chew I., Karunatilaka D., Tan C.P., Kalavally V. (2017). Smart lighting: The way forward? Reviewing the past to shape the future. Energy Build..

[36] Ruuvi Innovations Ltd. https://ruuvi.com/ruuvitag-specs/.

[37] Nordic Semiconductor Inc nRF52840 Dongle Mannual. https://www.nordicsemi.com/-/media/Software-and-other-downloads/Product-Briefs/nRF52840-Dongle-product-brief.pdf.

[38] Pierleoni P., Gentili A., Mercuri M., Belli A., Garello R., Palma L. (2021). Performance Improvement on Reception Confirmation Messages in Bluetooth Mesh Networks. IEEE Internet Things J..

[39] (2019). Bluetooth SIG Inc. Mesh Profile 1.0.1. https://www.bluetooth.com/specifications/specs/mesh-profile-1-0-1/.

[40] Kitchenham B., Brereton O.P., Budgen D., Turner M., Bailey J., Linkman S. (2009). Systematic literature reviews in software engineering–a systematic literature review. Inf. Softw. Technol..

[41] Xing L. (2020). Reliability in Internet of Things: Current status and future perspectives. IEEE Internet Things J..

[42] Brandt A., Buron J., Porcu G. (2010). Home Automation Routing Requirements in Low-Power and Lossy Networks. https://www.rfc-editor.org/rfc/rfc5826.

[43] Ibarra-Esquer J.E., González-Navarro F.F., Flores-Rios B.L., Burtseva L., Astorga-Vargas M.A. (2017). Tracking the evolution of the internet of things concept across different application domains. Sensors.

[44] Gubbi J., Buyya R., Marusic S., Palaniswami M. (2013). Internet of Things (IoT): A vision, architectural elements, and future directions. Future Gener. Comput. Syst..

[45] Jeon K.E., She J., Soonsawad P., Ng P.C. (2018). BLE beacons for internet of things applications: Survey, challenges, and opportunities. IEEE Internet Things J..

[46] Hassan W.H. (2019). Current research on Internet of Things (IoT) security: A survey. Comput. Netw..

[47] Balliu M., Bastys I., Sabelfeld A. (2019). Securing IoT Apps. IEEE Secur. Priv..

[48] Lacava A., Zottola V., Bonaldo A., Cuomo F., Basagni S. (2022). Securing Bluetooth Low Energy networking: An overview of security procedures and threats. Comput. Netw..

[49] Adomnicai A., Fournier J.J., Masson L. Hardware security threats against Bluetooth mesh networks. Proceedings of the IEEE Conference on Communications and Network Security (CNS).

[50] Krzysztoń M., Marks M. (2020). Simulation of watchdog placement for cooperative anomaly detection in bluetooth mesh intrusion detection system. Simul. Model. Pract. Theory.

[51] Álvarez F., Almon L., Hahn A.S., Hollick M. Toxic friends in your network: Breaking the bluetooth mesh friendship concept. Proceedings of the 5th ACM Workshop on Security Standardisation Research Workshop.

[52] Kaur N., Sood S.K. (2015). An energy-efficient architecture for the Internet of Things (IoT). IEEE Syst. J..

[53] Gotz J.D., Rayel O.K., Moritz G.L. (2021). Improving Bluetooth Mesh Energy Efficiency Using Clustering. J. Commun. Inf. Syst..

[54] Dunkels A. The ContikiMAC Radio Duty Cycling Protocol, SICS Technical Report T2011:13 ISSN 1100-3154. http://www.dunkels.com/adam/dunkels11contikimac.pdf.

[55] Hortelano D., Olivares T., Ruiz M.C. (2021). Reducing the energy consumption of the friendship mechanism in Bluetooth mesh. Comput. Netw..

[56] Romero E.A.D.L. (2019). Experimental Performance Evaluation of the Bluetooth Mesh Protocol for Monitoring Applications. Master’s Thesis.

[57] Sánchez R.C. (2018). Experimental Evaluation of Bluetooth Low Energy Mesh Networks. Master’s Thesis.

[58] Brandão A.S., Lima M.C., Abbas C.J.B., Villalba L.J.G. (2020). An Energy Balanced Flooding Algorithm for a BLE Mesh Network. IEEE Access.

[59] Ghamari M., Villeneuve E., Soltanpur C., Khangosstar J., Janko B., Sherratt R.S., Harwin W. (2018). Detailed examination of a packet collision model for bluetooth low energy advertising mode. IEEE Access.

[60] Perez-Diaz-de Cerio D., Hernandez-Solana A., Garcia-Lozano M., Bardají A.V., Valenzuela J.L. (2021). Speeding up bluetooth mesh. IEEE Access.

